# Is Toxin-Producing *Planktothrix* sp. an Emerging Species in Lake Constance?

**DOI:** 10.3390/toxins13090666

**Published:** 2021-09-17

**Authors:** Corentin Fournier, Eva Riehle, Daniel R. Dietrich, David Schleheck

**Affiliations:** 1 Microbial Ecology and Limnic Microbiology, University of Konstanz, 78457 Konstanz, Germany; corentin.fournier@uni-konstanz.de; 2 Human and Environmental Toxicology, University of Konstanz, 78457 Konstanz, Germany; eva.riehle@uni-konstanz.de; 3 Limnological Institute, University of Konstanz, 78457 Konstanz, Germany

**Keywords:** *Planktothrix*, *Synechococcus*, microcystins, temperate lakes

## Abstract

Recurring blooms of filamentous, red-pigmented and toxin-producing cyanobacteria *Planktothrix rubescens* have been reported in numerous deep and stratified prealpine lakes, with the exception of Lake Constance. In a 2019 and 2020 Lake Constance field campaign, we collected samples from a distinct red-pigmented biomass maximum below the chlorophyll-a maximum, which was determined using fluorescence probe measurements at depths between 18 and 20 m. Here, we report the characterization of these deep water red pigment maxima (DRM) as cyanobacterial blooms. Using 16S rRNA gene-amplicon sequencing, we found evidence that the blooms were, indeed, contributed by *Planktothrix* spp., although phycoerythrin-rich *Synechococcus* taxa constituted most of the biomass (>96% relative read abundance) of the cyanobacterial DRM community. Through UPLC–MS/MS, we also detected toxic microcystins (MCs) in the DRM in the individual sampling days at concentrations of ≤1.5 ng/L. Subsequently, we reevaluated the fluorescence probe measurements collected over the past decade and found that, in the summer, DRM have been present in Lake Constance, at least since 2009. Our study highlights the need for a continuous monitoring program also targeting the cyanobacterial DRM in Lake Constance, and for future studies on the competition of the different cyanobacterial taxa. Future studies will address the potential community composition changes in response to the climate change driven physiochemical and biological parameters of the lake.

## 1. Introduction

The formation of cyanobacterial blooms involves the complex interplay of regional and biological variables, and blooms have been reported worldwide with an increasing frequency [1,2]. Although it has been shown that climate change and eutrophication are, in many cases, major contributors to bloom formation, the mechanisms through which nutrients and temperature interact to amplify blooms varies extensively between cyanobacterial groups. Moreover, the optimal growth temperature and nutrient availability of cyanobacteria is species specific, making inferences from studies with other species more complex [2,3]. Indeed, different cyanobacterial species predominate depending on the N/P ratio [3]. The filamentous cyanobacterial genus, *Planktothrix*, occurs preferentially in prealpine and alpine lakes in temperate regions, and has been responsible for many blooms in the past (for example, in Lake Zurich [4,5], Lake Bourget [6], Lake Garda [7] and Lake Mondsee [8]), where its mass occurrence can evolve to become a major influence on the food web [9,10]. The success of *Planktothrix* spp. is attributed to its adaptability, including its ability to regulate buoyancy, and the use of phycobilins, in addition to chlorophyll-a [11,12], as chromophores. By virtue of the phycobilins, phycocyanin and phycoerythrin, *Planktothrix* spp. can absorb light in large parts of the electromagnetic spectrum; specifically, blue and green light, thus conferring its red appearance. Consequently, the free-floating *Planktothrix* spp. usually develop blooms at depths of 9–15 m [11,13]. Bloom depths within a stratified lake can be influenced by the internal waves, and can impact the growth of *Planktothrix* spp. by changing the light availability [14]. Similarly to many other cyanobacterial species, mass occurrences of *Planktothrix* spp. are supported by warmer water temperatures, mediating a more stable lake stratification in the summer [9,15]. Strikingly, *Planktothrix* spp. can, additionally, form prominent winter blooms and can thrive even beneath an ice cover, allowing for full year dominance [16,17]. Although microcystin production in winter blooms, on and under ice covers, has been reported, toxins appear to be less abundant in colder environments than in warmer environments [16,18,19].

The genus *Planktothrix* is currently distinguished in nine species, including red and green phenotypes, with the most prominent representatives being *P. rubescens* and *P. agardhii*, respectively, which mostly occur in the freshwater ecosystems of temperate regions [20]. Similarly to many other cyanobacteria, *Planktothrix* spp. are capable of producing microcystins (MCs), which are known to be toxic to humans, as well as other mammalian and non-mammalian species [15,16,17,18]. Hence, cyanobacterial blooms are often associated with the detection of increased intra- and extra-cellular toxin(s) [21]. Despite a plethora of efforts, neither the trigger for toxin production, nor the factors resulting in the development of toxic cyanobacterial blooms, have been elucidated. While toxins are suggested to be part of a defense mechanism against zooplankton or parasites [22,23,24], and toxin producing strains seem to have an advantage over non-toxic strains [25,26], the molecular ‘switch’ that turns toxin production on has not yet been discovered. Irrespective of the latter, the potential adverse impact of toxic cyanobacterial blooms on human health, society, economy and ecology highlights the importance of an improved understanding of cyanobacterial bloom formation, with or without concomitant toxin production [27,28].

Microcystins (MCs) share one common monocyclic structure with a molecular weight of approximately 1 kDa, which is composed of seven amino acids, (three *D*-amino acids, one *N*-methyldehydroalanine, two *L*-amino acids at the hypervariable positions two and four, and the unique amino acid ADDA (Figure S1) [29]). Variations in amino acid composition, and modifications such as methylations, create extreme structural diversity—at least 279 different congeners have been reported [30]. MC synthesis is encoded by 9–10 genes that constitute one *mcy* gene cluster [31]. The toxicity of MCs is induced by covalent binding, the inhibition of ser/thr protein phosphatases and the concomitant hyperphosphorylation of cellular proteins [32,33,34], whereby the toxicodynamically relevant biological availability of MCs highly depends on the route of exposure and the respective MC congener [35,36]. MC concentrations of <35 µg/L were reported in *Planktothrix* spp. blooms [37,38]. When considering the current World Health Organization (WHO) guideline value of 1 µg MC/L in drinking water [39], which translates to approximately 10 µg/L of raw water, largely depending on the type of water treatment used [40,41], it becomes obvious that toxin-producing *Planktothrix* spp. blooms must be taken as a serious threat to freshwater systems serving as drinking water resources.

Unlike other prealpine lakes, Lake Constance, a drinking water reservoir for more than four million inhabitants, has not yet seen prominent and recurring, well-documented blooms of *Planktothrix* spp. Although *P. rubescens* has the capability to dominate entire lake ecosystems, even at low nutrient concentrations (e.g., Lake Bourget [6]), analyses of Lake Constance samples revealed only low abundances of *Planktothrix* spp. to date [9,42]. Indeed, the first prominent appearance of *Planktothrix rubescens* was documented in 2016, when *P. rubescens* filaments were observed in various German, Austrian, and Swiss sampling sites at Lake Constance [42,43].

No molecular phylogenetic studies have been conducted, to date, to evaluate the composition of the red-pigmented cyanobacterial community in Lake Constance, in water depths that are typical for the blooms of *Planktothrix* spp. (i.e., below the chlorophyll-a maximum). In a field campaign in the Überlingen embayment of Lake Constance (47.7571° N 9.1273° E), we observed reddish colored plankton filters in the samples taken at 18 m water depth, in the summer of 2019. Subsequently, water samples were taken at the depths of maximal red pigment (phycoerythrin) concentration, as determined by a fluorescence probe, on each of the fortnightly sampling days, between June and October 2019, and between July and September 2020. The composition of the cyanobacterial community at this deep water red pigment maximum (DRM) was assessed through Illumina PCR-amplicon sequencing, using cyanobacteria-specific primers, while UPLC–MS/MS analyses were applied to determine the toxin concentrations. Potentially toxin-producing species were identified and quantified via quantitative PCR, using *Planktothrix*- and microcystin-biosynthesis-specific (*mcy*) primers, and Sanger sequencing.

## 2. Results

### 2.1. Blooms of Red-Pigmented Phytoplankton at Water Depths below the Chlorophyll-a Maximum in Lake Constance

**Figure 1:**
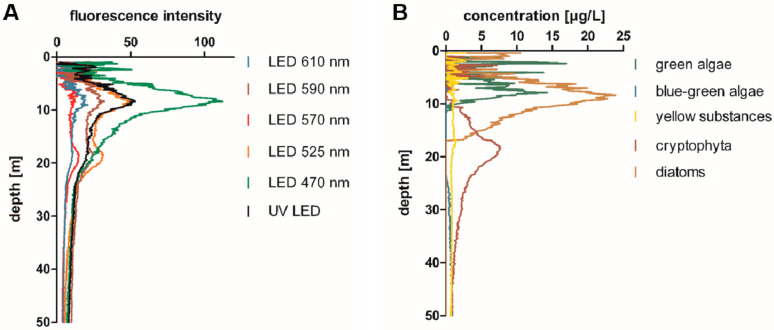
Representative depth profile recorded with a Moldaenke FluoroProbe multichannel fluorimeter, indicating the high abundance of red-pigmented biomass at a water depth below the chlorophyll-a maximum in Lake Constance on 1 July 2019. (**A**) Absolute fluorescence intensities recorded at the different excitation wavelengths. (**B**) Abundance of the different algae classes as attributed by FluoroProbe. Red-pigmented cyanobacteria are attributed to ‘cryptophyta’. In this example, the chlorophyll-a maximum was determined at 8–10 m water depth (Figure 1A, 470 nm), and a second maximum, indicating a red-pigmented biomass, at approximately 18–20 m water depth (Figure 1A, 570 nm; 1B, cryptophyta).

Multiwavelength fluorometer profiles were taken along the water column, using a Moldaenke FluoroProbe, and were evaluated directly on the ship to determine the depths of the maxima of green pigment (chlorophyll-a maximum; predominantly diatoms and green algae) and red pigment concentrations (deep water red pigment maximum, DRM). An example of a depth profile for all of the recorded fluorescence channels, as well as of the interpretation of the abundance of different algae classes (as calculated by the Moldaenke FluoroProbe) is depicted in Figure 1. An increased abundance of red-pigmented cyanobacteria, such as phycoerythrin-rich *Planktothrix* spp. or *Synechococcus* spp., was suggested by the elevated fluorescence intensity at 570 nm (and 525 nm) excitation wavelength (Figure 1). According to the distinction of algae classes used by the Moldaenke FluoroProbe, these phycoerythrin-rich algae were attributed to represent ‘cryptophyta’.

### 2.2. Phycoerythrin-Rich Synechococcus Phylotypes Dominated the DRM Cyanobacterial Community

In order to characterize the cyanobacterial community composition that is presumably present at the DRM, the plankton biomass at the DRM was collected on Whatman GF6 glass fiber filters. The filters appeared reddish, compared to the yellow-green filters obtained from the chlorophyll-a maximum sample (see Supplementary Material, Figure S2). The total DNA was extracted from the filters, and a fragment of the 16S rRNA gene was amplified using the cyanobacteria-specific primers, CYA359F and CYA784R [44]. Illumina sequencing, with 300 bp paired-end reads, was employed. These primers amplified cyanobacteria phylotypes, which allowed for the collection of phylogenetic information at a finer resolution, and also of the low abundant cyanobacteria at the DRM. Taxonomic affiliation was carried out using two different reference databases: SILVA_138 and Greengenes. The cyanobacteria taxonomy was consistent between both databases, with the exception of the *Synechococcus* genus in Greengenes (replaced by *Cyanobium*_PCC-6307 in SILVA; *Cyanobium*_PCC-6307 is a heterotypic synonym of *Synechococcus* sp PCC-6307).

**Figure 2:**
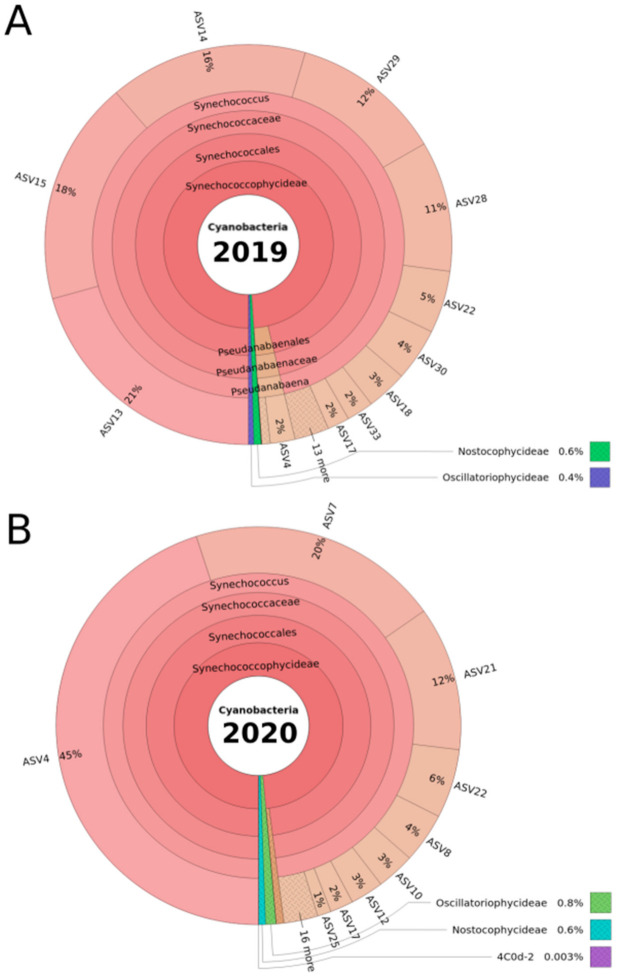
Krona plot of the DRM cyanobacterial community composition, as determined by 16S rRNA gene-amplicon sequencing across the sampling periods in 2019 (**A**) and 2020 (**B**). The community analysis was carried out by filtration of DRM water samples, total DNA extraction of the filters and PCR amplicon sequencing of the cyanobacteria-specific 16S rRNA gene region V3–V4 (380 bp length). For this Krona plot, and as an overview, the results shown are based on all samples combined per year. Taxonomic affiliation was carried out using the Greengenes reference database, and all taxonomic ranks are represented in the plot. Amplicon sequence variants (ASVs), as outputs of the Dada2 software package (see Section 5), distinguish sequence variations by a single nucleotide, giving ASVs a higher resolution than the operational taxonomic units (OTUs) typically used. Therefore, ASVs were used as the deepest taxonomic rank in our study. Total relative abundance was calculated by dividing the number of reads affiliated to an ASV in a sample by the total number of reads in the sample.

Subsequently to bioinformatic processing and the removal (filtering) of the extremely low abundant phylotypes (i.e., phylotypes represented by less than three reads in at least 20% of all samples; see Material and Methods), 35 amplicon sequence variants (ASVs) were detected in 2019, and 37 were detected in 2020 (Figure 2). Each ASV was affiliated at the level of either genus or order, depending on the last common taxonomic rank between the SILVA and Greengenes databases (note that species rank could not be affiliated by the amplicon sequencing technique that we used).

For both the 2019 and 2020 sampling campaigns, the genus *Synechococcus* clearly dominated the cyanobacterial DRM community (as examined using amplicon sequencing), occupying 96% (SILVA) or 98% (Greengenes) of the total relative read abundance, and representing 65.7% (SILVA) and 67.6% (Greengenes) of the detected ASVs in the community (see Supplementary Material, Table S2). For 2019, five *Synechococcus* ASVs represented 78% of the total relative abundance, with ASV13 being the most abundant with 21% total relative abundance (Figure 2A). For 2020, only three of the ASVs affiliated to *Synechococcus* contributed to 77% of the total relative abundance, with ASV4 contributing to almost half (45%) throughout the year (Figure 2B).

Although *Synechococcus* dominated the cyanobacterial community at the DRM in 2019 and 2020, each of the two ASVs that are affiliated to *Planktothrix* could be detected in both years. *Planktothrix* (Oscillatoriophycideae in Figure 2A,B), with 0.4% (2019) and 0.9% (2020) of the total relative abundance (Figure 2A,B), represented only low abundant taxa, together with Nostocophycideae. In addition, two *Microcystis* spp. ASVs were detected in 2019, at 0.06% of the total relative read abundance. Only one *Microcystis* spp. ASV was detected in 2020, with a very low total relative abundance of 0.008%. Although the respective relative abundances of *Planktothrix* and *Microcystis* species are low, the strong bioinformatic filtration (for details, see Section 5.6) confirms the biological significance of these amplicon sequencing results. The ASVs of the most abundant *Synechococcus* spp., as well as the *Planktothrix* spp. and *Microcystis* spp. ASVs, were used for further analyses.

### 2.3. Synechococcus Rubescens and Cyanobium Gracile Clusters in 2019 and 2020

**Figure 3:**
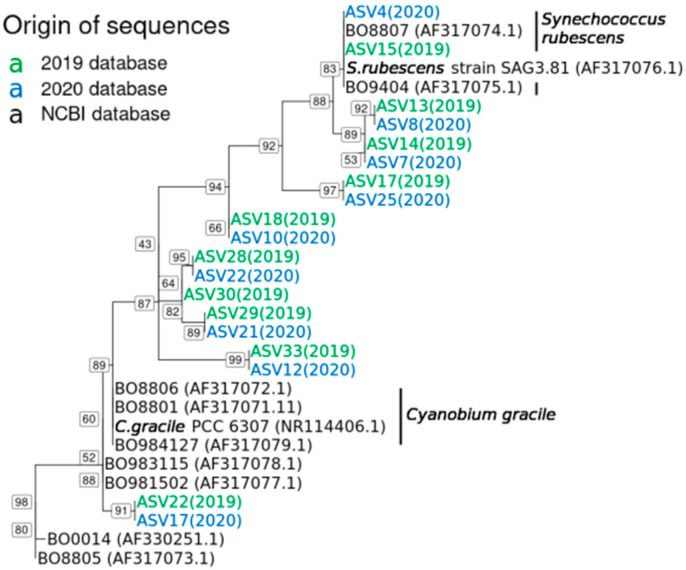
Illustration of the phylogenetic relationship of the *Synechococcus* spp. taxa observed in 2019 and 2020. The phylogeny is based on the cyanobacteria-specific V3–V4 (380 bp) 16S rRNA gene region. Colors correspond to the origin of the sequences, with green referring to ASVs observed in 2019 and blue referring to ASVs observed in 2020 (sampling year also in brackets). Sequences from *Synechococcus spp.* taxa, previously isolated from Lake Constance [45], and sequences of *Synechococcus rubescens* strain SAG3.81 and *Cyanobium gracile* PCC 6307 from NCBI were used as references (indicated in black). IQ-TREE was used for phylogenetic inference using maximum likelihood. The model of nucleotide substitutions used was TPM2+F + I [46], determined as the best-fit model by ModelFinder [47], based on the Bayesian information criterion scores and weights. Numbers at the internal nodes represent the percentage support of this specific node in 1000 bootstrap testing. The tree was rooted using the sequences of the cyanobacterium *Anacystis nidulans* PCC 6301 as an outgroup (not shown). The reference sequences are identified by their accession numbers in brackets.

We examined the phylogenetic relationship of the main *Synechococcus* ASVs that were detected in 2019 and 2020, with the reference sequences of (i) all the cultivated phycoerythrin-rich *Synechococcus* spp. of Lake Constance, as established by Ernst and colleagues in 2003 [45], and (ii) *Synechococcus rubescens* and phycoerythrin-rich *Cyanobium gracile* reference sequences, as established by NCBI (Figure 3). The relationship was established using the appropriate sequence fragments, representing the PCR amplicon of 380 bp, with the IQ-TREE program [46], based on a phylogenetic inference using the maximum likelihood, coupled with ModelFinder to determine the best-fitting nucleotide substitution model [47]. Although the target sequence was shorter than the full 16S rRNA gene sequences established by Ernst et al., 2003, the phylogenetic relationship between the reference sequences remained the same, thereby confirming our analyses. The ASVs from this study were always grouped in pairs, with one ASV from 2019 (Figure 3, green font) and another from 2020 (Figure 3, blue font), as a reflection of the reoccurring *Synechococcus* phylotypes across the two years, further confirming our analysis.

Overall, the ASVs were grouped into two main clusters, either more closely related to the *S. rubescens* or to the *C. gracile* reference sequence (Figure 3). The top three most abundant ASVs for 2019 (ASV13, ASV14 and ASV15; see Figure 2 and below) and the top two most abundant for 2020 (ASV4 and ASV7; Figure 2), were more closely related to the *Synechococcus rubescens* NCBI reference sequence. For this group, two reference sequences of Lake Constance phycoerythrin-rich *Synechococcus* isolates [45] were available (Figure 3; BO8807 and BO9404), while for the *C. gracile* group, the reference sequences of seven Lake Constance isolates were available (Figure 3).

The megablast results were analogous to the phylogenetic tree shown, with the same ASVs in 2019 and 2020 being affiliated more closely to either *Synechococcus rubescens* or *Cyanobium gracile* (Supplementary Material, Table S3a,b), with the exception of ASV18 in 2019 and ASV10 in 2020. These ASVs showed identical percentage identities and E-values in the megablast for both *S. rubescens* and *C. gracile* (Table S3a,b) and were, therefore, grouped in between both clusters (Figure 3).

### 2.4. Dynamics of Synechococcus ASVs in 2019 and 2020

**Figure 4:**
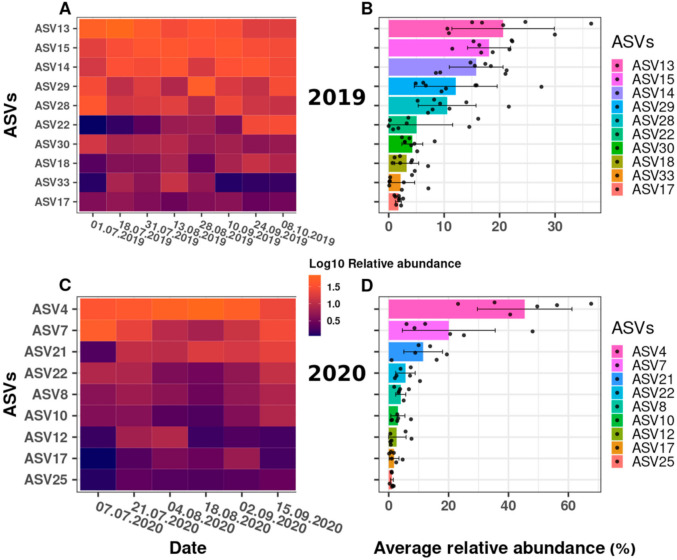
Dynamics of relative abundance changes for the *Synechococcus* ASVs observed during the 2019 and 2020 sampling campaigns. Shown are heatmaps across all sampling dates (**A**,**C**) and the ASVs’ average relative abundances (**B**,**D**) for each year (bars) with each individual data point indicated (grey dots). For the y-axes of heatmaps (**A**,**C**), and average relative abundances (**B**,**D**), the different ASVs were ordered from the most abundant (top) to the least abundant (bottom). The heatmap data was log10 transformed from the read count matrix, and, to avoid introducing infinity for zero read counts, we added one artificial read to every cell prior to log10 transformation (log10[x + 1]). Color schemes vary between dark blue for low log10 values to bright orange for high log10 values; a higher log10 value means a higher relative abundance. Please note that the x-axes for B and D do not have the same scale. The average relative abundance across all samples of each year was calculated as it was for the Krona plot (see Figure 2). Standard deviation was also calculated and represented for each ASV; if the graphical representation of the standard deviation was below 0, the minimum error bar value was set to 0.

We examined the change in the relative abundance of the *Synechococcus* ASVs detected, over time. Figure 4 illustrates the dynamics of the *Synechococcus* ASVs as a heatmap, using relative abundance data after the log10 transformation (Figure 4A,C) and the data distribution (Figure 4B,D) as the mean and standard deviation of the relative abundances in a combined bar and jitter plot. Each dot represents the relative abundance of the taxa at a specific date (relative abundance values (%) per ASV across the sampling dates are shown in Supplementary Material, Table S4a,b).

Overall, the observed changes in the phylogenetic structure across the sampling dates suggested a high degree of successional change within the *Synechococcus* spp. community at the DRM. Some ASVs varied from being almost undetectable in the beginning to a relative abundance maximum later in the year, or showed the opposite trend (being abundant at the beginning or in the middle of the sampling campaigns), while other ASVs showed a comparatively stable (and low) relative abundance across the sampling campaigns. Indeed, while 10 ASVs were registered in 2019, only nine ASVs were registered in 2020 (Figure 4A,C). Thus, different ASVs appeared to dominate, and, therefore, provide a successional change, in the two field campaigns. For example, ASV13, as the most prominent ASV in 2019 (Figure 4B), reached its maximum in July (up to 36%; Figure 4A) and decreased thereafter in its relative abundance (to approximately 10%) at the end of September, while the two second most abundant ASVs in 2019 (ASV 14 and 15; Figure 4B) reached their maxima in mid-August and later in the year (see Figure 4A and Table S4a). Similarly, in 2020, ASV7 represented almost 50% of the total relative abundance in early July, but decreased to approximately 6% by mid-August, while the most abundant taxon in the 2020 sampling campaign (ASV4; Figure 4C,D) reached its peak at the end of August (68%) and decreased to approximately 23% by the end of the campaign (Figure 4C). Furthermore, the third most abundant ASV21 in 2020 represented only approximately 1% of the total relative abundance at the beginning of the sampling campaign, but almost 20% at its end (see Figure 4C, Table S4b).

The statistical relevance of the differences in *Synechococcus* ASV relative abundance was confirmed by a Kruskal–Wallis analysis of variance, with a *p*-value far below the threshold of 0.05 (*p*-value of 3.95 × 10^−10^ and 1.27 × 10^−5^ for 2019 and 2020, respectively). In an attempt to group the taxa according to their relative abundance differences, a post hoc Conover–Iman test was performed using a Benjamini Yekutieli *p*-value adjustment method for False Discovery Rate control (Table S5a,b). For 2019, the results indicated two groups. First, a group comprising ASVs that comparatively stably dominated the cyanobacterial community at the DRM throughout July and October (together 63% to 87% of the total relative abundance); these were ASV13, 14, 15, 28 and 29. A second group statistically differed from the first group (i.e., ASV17, 18, 22, 30 and 33), for which the abundance differences appeared to be larger and/or occurred within shorter time intervals: For example, ASV22 reached its high relative abundance of 15% on only two sampling dates (in September and October, see Figure 4A and Figure S4a), as also discussed above. For 2020, the Conover–Iman test did not significantly separate the ASVs into two groups as for 2019 (Table S5b), although visually (Figure 4D), ASV4 dominated the cyanobacterial community with an average relative abundance of 45% throughout 2020.

### 2.5. Dynamics of Planktothrix and Microcystis ASVs, the Abundance of Microcystin Biosynthesis Genes and the Concentration of Microcystins in Samples Taken during 2019 and 2020

**Figure 5:**
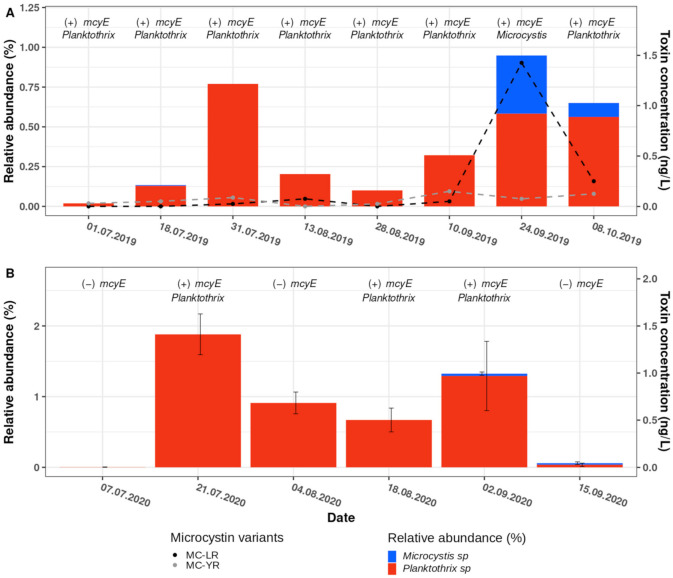
Relative abundance of the *Planktothrix* and *Microcystis* ASVs observed in 2019 and 2020, and of microcystin concentrations for samples taken in 2019. The bar plots represent the total relative abundance observed for the two *Planktothrix* ASVs (red bars) detected in 2019 (**A**) and 2020 (**B**), for the two *Microcystis* ASVs (blue bars) detected in 2019, and the single *Microcystis* ASV detected in 2020. The *x*-axes represent the different sampling dates and the left *y*-axis the relative abundance (%). The error bars in B represent the standard deviation of the relative abundance, as calculated from biological triplicates (n = 3); no error bars are represented for 2019, as only one sample was collected per sampling date. The right *y*-axis represents the toxin concentration (ng/L) determined for independently collected DRM filters, for which the microcystin variant concentrations are indicated by dashed lines in black (MC-LR) and grey (MC-YR) (see main text). The text on top of each bar indicates the results of the PCR amplification of the microcystin synthesis gene *mcyE* (+, detected; −, not detected) and of the phylogenetic affiliation of the *mcyE* consensus sequence to either *Planktothrix* or *Microcystis*, as established by Sanger sequencing of the PCR amplicons (see main text).

In both the 2019 and the 2020 amplicon sequencing datasets, we detected two ASVs affiliated to the *Planktothrix* genus. The relative abundance of *Planktothrix* ASVs in 2019 increased from July to late September, with a peak on 31 July, where 0.75% of the total cyanobacterial community were *Planktothrix* ASVs (Figure 5A). Likewise, in 2020, *Planktothrix* ASVs were abundant from the end of July to the end of September, with a maximum of approximately 2% relative abundance on 21 July (Figure 5B). While one of the *Planktothrix* ASVs was affiliated to *Planktothrix rubescens* (a toxin-producing *Planktothrix* species), the amplicon sequencing of the cyanobacteria-specific 16S rRNA-gene fragments of the other ASVs did not allow for taxonomical distinction at the species level (i.e., between the mostly non-toxin-producing *P. agardhii* and the toxin-producing *P. rubescens*). For example, a megablast alignment of the *Planktothrix* ASVs suggested *Planktothrix agardhii* and *Planktothrix rubescens* were the top hits, with identical query coverage and E-values, and percentage identities varying between 99.70% and 100% for *P. agardhii*, and 99.38% to 99.74% for *P. rubescens*.

Beyond the *Planktothrix* ASVs, two ASVs that are affiliated to the *Microcystis* genus were detected in 2019; however, only one *Microcystis* ASV was detected in 2020. As both genera, *Planktothrix* and *Microcystis*, are renowned for their toxin producing species, more in depth analyses were carried out regarding the toxin producing potential of the species found in the Überlingen embayment.

To confirm the detection of the *Planktothrix* genus in Lake Constance at very low levels, particularly when compared to *Synechococcus*, quantitative PCR (qPCR) was performed to estimate the abundance of toxin-producing *Planktothrix* genotypes. Specifically, we used *Planktothrix*-specific primer pairs for the 16S rDNA gene and the *mcyBA1* gene, as described by Ostermaier and Kurmayer, 2009 (Table 1, [48]). *Planktothrix mcyBA1* encodes for the first adenylation domain in non-ribosomal peptide synthase (NRPS) gene clusters, and is present only in species that are capable of toxin production. Briefly, we created a standard curve using a dilution series of *Planktothrix* DNA (0.00001–100% *Planktothrix* DNA diluted in *Microcystis* DNA) and then calculated the relative abundance of *Planktothrix* DNA in our samples using a linear regression. The calculated relative abundance of *Planktothrix* ranged between 0.1 and 0.6% in 2019, and 0.1 and 0.01% in 2020 (Figure S3). Although the relative abundance calculated by qPCR differed from the relative abundances that were found with amplicon sequencing (Figure S3 and Figure 5), the trend aligned well between both methods. Statistical analyses of the differences between the 16S-rRNA gene and *mcyBA1* amplifications (ANOVA, see Ostermaier and Kurmayer, 2009) showed no significant difference in abundance with respect to toxin-producing or non-toxin-producing *Planktothrix* genotypes, suggesting that there was only one genotype of *Planktothrix* present in 2019 and 2020. To confirm the presence of toxin-producing cyanobacterial species in Lake Constance, we used universal *mcyE*-specific PCR primers (HEPF/R, Table 1, [49]). Being a member of the MC production gene cluster, *mcyE* is partly responsible for the synthesis of the ADDA chain in microcystins, as well as the incorporation and synthesis of *D*-Glu [50]. For the 2019 sampling campaign, the PCR yielded amplicons for every sample tested, suggesting the presence of potential microcystin producers throughout the year. The subsequent Sanger sequencing of the PCR products, and analysis of the consensus sequences with megablast, attributed toxin-producing capabilities to *Planktothrix* species (Figure 5A), except for the sample taken on 24 September 2019, where the *mcyE* consensus sequence had the highest alignment scores with *Microcystis*, thus matching the date with the highest relative abundance of the *Microcystis*-affiliated ASV. For 2020, *mcyE* amplicons were observed less consistently than in 2019, although, as seen in 2019, Sanger sequencing of these amplicons attributed the toxin-producing capabilities to *Planktothrix* spp. (Figure 5B).

Collectively, we provide evidence that toxin-producing *Planktothrix* spp. (and/or *Microcystis* spp.) are present in the Überlingen embayment of Lake Constance. However, our data did not allow us to conclude whether or not toxin production took place during the sampling campaigns, as many species can carry the gene cluster without actively producing the toxins [51]. Consequently, we analyzed the biomass samples that were collected independently on filters from the DRM for microcystins, using UPLC–MS/MS (Figure 5A,B). Microcystin concentrations peaked at the end of September 2019, where, in total, approximately 1.5 ng/L of intracellular microcystins were found. The microcystin variants present were MC-LR (leucine and arginine in hypervariable region) and MC-YR (leucine and tyrosine in hypervariable region), with MC-LR being almost solely responsible for the peak in toxin concentration in September 2019 (Figure 5A). Strikingly, for the samples collected during 2020, no toxins were detected under the conditions we used.

### 2.6. Retrospective Evaluation of Depth Profiles for the Lake Überlingen Routine Sampling Site

During our sampling campaigns in 2019 and 2020 (and the ongoing 2021 campaign), prominent DRM were observed from June onwards, particularly after long and stable good weather periods. This is best illustrated on 1 July 2019, when we observed a first prominent DRM at 18.4 m depth at the routine sampling site ‘Wallhausen’, in the Überlingen embayment of Lake Constance. This DRM appeared after the weather presented a stable window of approximately two weeks with predominant sunshine and no precipitation, low wind and elevated temperatures, as depicted in Figures S5, S7 and S8.

**Figure 6:**
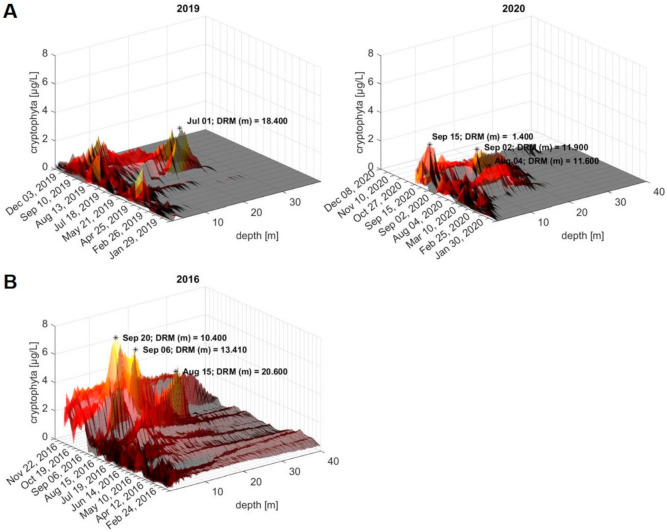
Depth profiles recorded with the FluoroProbe across the sampling campaigns in 2019 and 2020 (**A**), in comparison to the year 2016, in which *P. rubescens* blooms were reported in Lake Constance (**B**). Depicted are the FluoroProbe profiles for ‘cryptophyta’ abundance (cf. Figure 1) (expressed in µg chlorophyll-a per liter), recorded as proxy of red pigment abundance, in the water column from 0–40 m depth at the routine sampling site ‘Wallhausen’, in the Überlingen embayment of Lake Constance. Prominent DRM are highlighted with dates and water depths. Coordinates of the study site: 47.7571°N 9.1273°E; for an illustration, see Figure S4. In 2016, blooms of *P. rubescens* were reported for Lake Constance in September–October at various sampling sites (i.e., of the German, Austrian and Swiss sections of Lake Constance [42,43]). For 2016, FluoroProbe data is available for the Überlingen embayment of Lake Constance (**B**).

Three-dimensional (3D) plots of the FluoroProbe depth profiles for ‘cryptophyta’ content, as proxy, in the water column at 0–40 m depth (Figure 1) across the sampling campaigns in 2019 and 2020 are depicted in Figure 6A. Furthermore, we retrospectively evaluated the ‘cryptophyta’ depth profiles that were collected in the previous years from the routine sampling site, and transformed these into 3D plots using MATLAB (for years 2009–2018, see Supplementary Material, Figure S6). During the last twelve years, DRM have occurred multiple times (Figure S6, plots from 2011 and 2015–2020). Specifically, in 2016, we observed a prominent DRM in the summer, with maximum ‘cryptophyta’ concentrations of 6 µg/L on 6 September and 20 September (Figure 6B). Corresponding to the DRM, the high abundance of *Planktothrix rubescens* has been reported in various sampling sites at Lake Constance in 2016 [42,43].

Overall, prominent DRM were observed from July to October, with a maximum ‘cryptophyta’ content of approximately 2–4 µg/L (as estimated based on the FluoroProbe calibration), and at water depths ranging between 10 and 20 m (Figure 6A and Figure S6). The observed variation of the DRM depths likely follows the lake’s internal waves, as previously found in Lake Ammer in Germany and Lake Bourget in France [14,52]. Although we could speculate that these peaks represent high abundances of *P. rubescens* in the Überlingen embayment, the absence of any appropriate samples allowing for DNA or toxin analyses available from that time preclude any corroboration.

## 3. Discussion

The characterization of the recurring blooms of red-pigmented cyanobacteria in the Überlingen embayment of Lake Constance at water depths of 15–20 m by amplicon sequencing demonstrated the presence of *Synechococcus*, *Planktothrix* and *Microcystis*. Briefly, the relative abundance data suggested that *Synechococcus* taxa predominated the community (96–98%) at these water depths, while *Planktothrix* and *Microcystis* taxa were detectable only in very low abundances. For example, in 2020, up to 45% of the total relative abundance was represented by a single *Synechococcus* ASV (ASV4; Figure 2B and Figure 4C,D). Moreover, the observed changes in the relative abundance of *Synechococcus* ASVs across the sampling dates suggest a high degree of successional change within the *Synechococcus* spp. community at the DRM (Figure 4).

Phycoerythrin-rich *Synechococcus* species from Lake Constance have been investigated previously [45,53,54], and, similarly to *Planktothrix* spp., they can express a large variety of phycobilins, thereby exploiting diverse light conditions [45,55]. Interestingly, the highest abundant *Synechococcus* taxa found in our studies in 2019 and 2020 are closely related to either *S. rubescens* or *C. gracile*, forming two main clusters (Figure 3, Table S3a,b). For the 2019 and 2020 sequences, a discrimination between *S. rubescens* and *C. gracile* is difficult (Figure 3), as the 380-bp PCR amplicon used in this study is cyanobacteria-specific, and allows us to affiliate taxa with high confidence only up to the genus rank. However, each *Synechococcus* taxon detected in 2019 is paired with a taxon detected in 2020, and their sequence alignment showed a 100% identity with no gap, suggesting recurring phylotypes/ecotypes, at least across the two years. The close association of our sequences with those from the early 2000s [45] suggests a stable deep water cyanobacterial community in Lake Constance. The latter interpretation is supported by the earlier finding that different lineages of *Synechococcus* spp. can adapt to, and thrive in, specific ecological niches [56].

The predominance of *Synechococcus* species over other cyanobacterial genera was reported for the experimental cocultures of *Synechococcus* and *Microcystis* strains, as well as varying phosphate and nitrogen concentrations [57,58]. Indeed, at low nutrient concentrations, which could also mirror the currently oligotrophic conditions of Lake Constance [59], *Synechococcus* outcompeted *Microcystis* in growth rate and final biomass [57,58]. The predominance of *Synechococcus* may be explained by its seemingly higher affinity for orthophosphates, more efficient nutrient uptake (due to a larger surface-to-volume ratio), as well as by the potential for competitive inhibition via the quorum sensing/quenching molecules between the two strains [57,58,60,61]. The latter laboratory findings were also corroborated in natural habitats, at least in regards to their trend [62].

Despite the seemingly stable predominance of *Synechococcus* in the deep water cyanobacterial community observed in Lake Constance over the last two decades [45], the de novo occurrence of *Planktothrix* spp. in 2016, and the reconfirmation of this earlier finding with our samples in 2019 and 2020, could suggest that *Planktothrix* spp. is in the process of establishing a stable presence in the Überlingen embayment of Lake Constance. The latter is of critical importance as mass occurrences of toxin-producing cyanobacteria at water depths of >20 m could become a threat to the water intake for the Sipplingen water treatment plant (https://www.bodensee-wasserversorgung.de, accessed on 7 May 2021 [63]) which serves >4 million people with drinking water. Indeed, we detected a low abundance of potentially MC-producing *Planktothrix* spp. using amplicon sequencing, as well as through *Planktothrix*-specific qPCR for 2019 and 2020 (Figure 5 and Figure S3). Furthermore, the presence of the microcystin biosynthesis gene cluster, *mcy*, was detected through the PCR amplification of *mcyE* (Figure 5). The subsequent Sanger sequencing of these amplicons identified *Planktothrix* spp. to be the main contributor to this gene sequence in our samples, with the exception of 24 September 2019, when the amplified *mcyE* consensus sequence identified the highest alignment scores in *Microcystis* spp. In *Planktothrix* spp., the *mcy* gene cluster can be inactivated by various mutations, including insertions or deletions, and, thus, non-toxic strains can develop [64]. Non-toxic strains are less successful in competition than their toxic relatives, and, thus, *Planktothrix* blooms are usually dominated by toxic strains [48]. Corresponding to the presence of *mcyE* in 2019, low amounts of the microcystins MC-LR and MC-YR were detected using UPLC–MS/MS. In 2020, although *mcyE* amplicons were detectable in some of our samples, concentrations of microcystins were below the detection levels of the UPLC–MS/MS that was used (LOD of UPLC–MS/MS method: 0.5 ng/mL [65]; therefore, the resulting LOD in our water samples: 62 pg/L lake water). Despite the low abundance of *Planktothrix* spp. and the low concentrations of detected toxins, the question does arise concerning whether the latter is a sign of a fundamental change in water body dynamics in the Überlingen embayment of Lake Constance (e.g., resulting from global warming). Indeed, the greater and prolonged stratification of water bodies, in conjunction with lowered nutrient levels, would promote a deep water euphotic ecosystem encompassing low-light specialized species, such as the picocyanobacterial *Synechoccocus* spp. and *Planktothrix* spp. [9,15], as was reported for other prealpine lakes (e.g., Lake Zurich [4], Lake Mondsee [8] and Lake Bourget [6]).

As *Planktothrix* spp. are stimulated by increased temperatures [15], the continuous shift toward higher overall temperatures could favor the perseverant establishment of toxin-producing *Planktothrix* spp., to the disadvantage of today’s *Synechococcus*-dominated DRM ecosystems. Considering that *Synechococcus* spp. are currently markedly outcompeting other cyanobacterial species occurring at these water depths, this may suggest that the allelopathic compounds from *Synechococcus* spp. can have an adverse effect on co-occurring species. Indeed, such effects have been observed for freshwater *Synechococcus* spp., which were able to impact the growth of other freshwater cyanobacteria or green algae [66,67]. Adverse effects caused by allelopathic compounds from marine *Synechococcus* spp. were also observed on various marine invertebrates [68], as well as other bacterial species [60]. In conclusion, the described effects indicate the widespread production of allelopathic compounds by *Synechococcus* species that can even influence other bacteria, plants and invertebrates.

In most cases, the co-occurrence of species occupying the same, or similar, ecological niche leads to the dominance of one species, largely depending on the individual species’ competitive advantages (e.g., nitrogen fixation, uptake of inorganic phosphorus, regulation of buoyancy, allelopathic compounds, etc.), as also shown by Weisbrod et al. [69]. This suggests that, if *Synechococcus* outcompetes *Planktothrix* in its ecological niche, allelopathic compounds (such as toxins), amongst other factors, may be used to compete against the respective other species. Indeed, allelopathic activity is one of the major competitive strategies of freshwater *Synechococcus* against coexisting phytoplankton species [66], further supporting the potential role of picocyanobacterial exudates in competition with other cyanobacteria, such as *Planktothrix*. Counterintuitively, some freshwater *Synechococcus* spp. possess a positive allelopathic activity towards *Microcystis* spp., and no effect on *Phormidium* spp., whereby *Phormidium* spp. (like *Planktothrix* spp.) is part of *Oscillatoriaceae* [66], suggesting that *Synechococcus* spp. could even promote the growth of toxin-producing species. The complex interplay of species in competition in Lake Constance emphasizes the need for further studies regarding the co-occurrence and dominance of *Synechococcus* spp., relative to *Planktothrix* and *Microcystis* spp. in this lake.

The geographical setting of the Überlingen embayment of Lake Constance, with its minor wind influence (Figures S7 and S8), is considered an additional factor that could favor the continuous development of deep water *Planktothrix* spp. populations. Indeed, light winds and convective mixing are highly important in the seasonal cycling of *P. rubescens* communities within a strongly stratified medium-sized lake [70]. In consequence, this means that toxin-producing *Planktothrix* spp. could possibly establish themselves as a dominant cyanobacteria species at deeper water levels, and may become a relevant concern for the quality of the Überlingen embayment of Lake Constance as a drinking water resource in the not too distant future.

## 4. Conclusions

Our study characterized the deep water red pigment maxima (DRM) in the Überlingen embayment of Lake Constance in 2019 and 2020 as being dominated by phycoerythrin-rich picocyanobacterial; namely, *Synechococcus rubescens* and *Cyanobium gracile.* Unlike other prealpine lakes, the DRM in Lake Constance is not dominated by the phycoerythrin-rich, filamentous and often toxin-producing *Planktothrix rubescens*. Indeed, the alignment of our results with the sequences from the Ernst et al. study (2003) demonstrates high sequence similarity, and suggests that the same species have been dominant at 15–20 m depth in Lake Constance for the past 20 years. However, we confirmed the reports from 2016 that *Planktothrix* spp. does occur in Lake Constance in the years 2019 and 2020, albeit at very low relative abundances. Nevertheless, microcystin concentrations of up to 1.5 ng/L were detected through UPLC–MS/MS in 2019, which appeared to be produced by *Planktothrix* and/or *Microcystis* spp. Hence, at present, Lake Constance seems to have a rather stable deep water cyanobacterial ecosystem, predominated by *Synechococcus* spp., although the geographical setting, as well as the continued climate warming, could favor the development and steady predominance of toxin-producing *Planktothrix* spp. This highlights the importance of a future monitoring program for Lake Constance, with emphasis on sequencing-based cyanobacterial community studies and microcystin monitoring, as well as the importance of competition studies regarding the different cyanobacterial taxa in relation to the physiochemical and biological parameters of the lake, particularly in respect to the ongoing climate change. Monitoring programs and hypothesis-driven competition studies may provide the required database to predict future deep water mass occurrences of toxin-producing cyanobacteria and, thus, help to secure Lake Constance as the drinking water resource for millions of people in the future.

## 5. Materials and Methods

### 5.1. Sample Collection

Samples were taken every two weeks from 1 July to 8 October in 2019, and 7 July to 15 September in 2020. The initial sampling in 2019 included only one replicate/day, while samples were collected in biological triplicates (n = 3) during 2020. A bbe Moldaenke FluoroProbe (FP) (SN: 01709; recalibrated at bbe in 2009, 2012, 2016 and 2019) was used to determine the deep red maximum (DRM). Samples were collected at that depth every other week in Upper Lake Constance (47.7571° N 9.1273° E), using a messenger-released Free Flow Water Sampler 5 L (HYDRO-BIOS, Altenholz, Germany). An overview of the samples taken during this study is illustrated in Table S1. Water was filtered through a 180 µm nylon net filter to exclude zooplankton and larger particles. Two liters of water were then filtered on one Whatman GF6 glass fiber filter while applying 2 bars pressure for the collection of plankton biomass. Filters were stored at −20 °C until analysis. This sampling method was applied to DNA extractions in 2019, and toxin extractions in 2019 and 2020. In 2020, DNA samples were collected independently (n = 3) on 0.2 µm polycarbonate filters.

### 5.2. DNA Extraction and PCR

DNA from the 2019 samples was extracted using the ZYMO Research Fecal/Soil/Microbe Microprep kit, following the manufacturer’s instructions. DNA of 2020 samples was extracted using a phenol/chloroform/isoamylalcohol protocol, adapted from Rusch et al., [71] and the JGI protocol [72]. Standard PCR was performed with Taq polymerase, using 2X Taq MasterMix (NEB) and 30 cycles. The microcystin synthesis gene, *mcyE*, was amplified with the HEPF/R primer set [49], and DNA from cultured *Microcystis aeruginosa* strain 78 was used as a positive control for the presence of *mcyE*. Planktothrix-specific primer pairs PcPI+/− (PC-IGS) and peamso+/− (*mcyA*) [8] were used for additional PCR reactions, with *Microcystis aeruginosa* strain 78 genomic DNA as a negative control, and *Planktothrix rubescens* strain 101 genomic DNA as a positive control.

### 5.3. RT-PCR/TNA

Quantitative Taq Nuclease Assays (TNA or TaqMan PCR) were performed with primers specific to *Planktothrix* spp. [48]. We quantified both *Planktothrix*-specific 16S rDNA and *mcyBA1*, which encodes the first adenylation domain of *mcyB*, and is indicative of all genotypes containing the *mcy* gene cluster. Both probes contained 5′ FAM as a fluorescent marker and 3′ TAMRA as a quencher dye. Each reaction contained 50 ng template DNA, 200 nM of primers and probes, and KAPA probe fast MasterMix. Amplification and quantification were carried out in triplicates in a Bio-Rad CFX96 cycler with the following protocol: 10 min at 95 °C to activate the hot start polymerase, followed by 50 cycles of 15 s at 95 °C, 60 s at 51.5 °C for 16S, 60 s at 57 °C for *mcyBA1*, 60 s at 68 °C and a final elongation for 5 min at 68 °C. The standard curve contained *Planktothrix* DNA mixed with *Microcystis* DNA in different ratios (0.00001–100% *Planktothrix* DNA diluted in *Microcystis* DNA). Data analysis using linear regression from the standard curve was performed using BioRad CFX manager, Microsoft Excel and GraphPad Prism 5.

### 5.4. Sanger Sequencing

Sanger sequencing was performed with the amplicons produced by the HEPF/R primer pair to identify the main microcystin producer in the samples. PCR amplicons were purified using a QIAquick PCR cleanup kit and sent to Eurofins Genomics for analysis. The identity of the obtained sequences was determined using Nucleotide BLAST megablast [73].

### 5.5. Amplification and Illumina Sequencing

Amplification of the V3–V5 hypervariable regions, and the cyanobacterial-specific V3–V4 hypervariable regions of the 16S rRNA gene, was performed with 0.02 U/µL of Phusion High Fidelity DNA polymerase, 1X Phusion HF Buffer, 200 µM of dNTPs (New England Biolabs, USA) and 0.5 µM of each primer. Primers targeting the V3–V5 hypervariable regions were 357F and 926R [74,75]. Cyanobacteria-specific primers were CYA359F and CYA784R [44]. Each PCR comprised an initial denaturation step of 3 min at 98 °C, followed by 30 cycles of denaturation for 45 s at 98 °C, annealing for 20 s at 62.4 °C (V3–V5) or 60 °C (cyanobacteria-specific) and extension for 8 s at 72 °C, and a final extension step for 5 min at 72 °C. Extracted DNA was added at a final concentration of 0.12 ng/µL. No purification step was performed; the PCR products were directly sent to Eurofins Genomics for sequencing using Illumina MiSeq 2 × 300 bp with the Microbiome Profiling Indexing only package. The data presented in this study are accessible on NCBI under the bioproject number PRJNA727470.

### 5.6. Bioinformatics Pipeline

The analysis was carried out using the already merged dataset provided by Eurofins Genomics, Konstanz, Germany. The expected fragment sizes were 569 bp for the V3–V5 amplicon and 425 bp for the V3–V4 cyanobacterial-specific amplicon. Reads were first trimmed using Trimmomatic [76], removing all reads with a Phred quality below 3 for the start and the end of the reads, below an average quality of 10 on a window of 3 base within the reads, and below a size of 500 bp for the V3–V5 amplicons and 380 bp for the V3–V4 amplicons. FastQC was used for quality control of the reads before and after trimming [77]. The following steps were performed using QIIME2 2019.10 [78]. Filtration of chimeras using the consensus method, denoising and dereplication of the quality reads were performed using the denoise and dereplicate single-end sequences (Dada2, denoise-single) and a reads learn of 2,000,000 reads for the training error model [79]. Quality trimming had already been performed using Trimmomatic, so no trimming step was performed here. Taxonomic affiliation was achieved using the classify-consensus-vsearch program and the databases SILVA_138 and Greengenes, with a percentage identity of 80%, 90%, 97% and 100%. Taxonomic results were merged and the highest percentage identity taxonomic affiliation was retained. Reference databases were previously trained using the feature-classifier extract-reads script with the sequences of the primers used for the amplification of the hypervariable regions. After training, the databases only contained the part of interest of the 16S rRNA gene for taxonomic assignation, V3–V5 hypervariable regions for the general 16S rRNA gene amplification and V3–V4 hypervariable regions for the cyanobacteria-specific primers. Taxonomy was mostly consistent between the SILVA_138 and Greengenes databases.

### 5.7. Phylogenetic Analysis

A megablast (highly similar sequence) was performed on the sequences affiliated to the genera *Planktothrix*, *Microcystis* and the most abundant *Synechococcus* using the 16S rRNA sequences reference database on NCBI. The top ten hit sequences and the description table were extracted. The *Synechococcus* sequences from Lake Constance analyzed and sequenced by Anneliese Ernst [45] were downloaded from the NCBI database. Two other 16S rRNA gene sequences, belonging to *Synechococcus* *rubescens* and *Cyanobium gracile*, were also collected from NCBI and used as reference for the phylogenetic analysis, as previously carried out by Anneliese Ernst. The collected dataset was then compared to the most abundant *Synechococcus* sequences in 2019–2020. Sequences were merged with our sequences into a fasta file and aligned using SeaView [80]. IQ-TREE 2.1.2 [46] was used to calculate the phylogenetic tree by maximum likelihood using 1000 bootstrap parameters. The model of nucleotide substitution used was TPM2+I+F [46], determined as the best fit model by ModelFinder [47], based on the Bayesian information criterion scores and weights. The tree was visualized on R using the package ggtree [81].

### 5.8. Statistical and Network Analysis

Statistical analysis was performed with R software [82], using the package Phyloseq [83], vegan [84] and visualized with the package ggplot2 [85]. Features represented by less than 3 reads in below 20% of the samples were discarded. Chloroplast-affiliated features were removed from both datasets, bacteria-affiliated taxa were also removed from the cyanobacterial-specific dataset only. No rarefaction has been applied to the dataset [86]. After filtration, the lowest number of reads in a sample was 19,175 for the cyanobacteria-specific dataset and 24,329 for the general 16S rDNA dataset in 2019. The 2020 dataset consisted of only the cyanobacteria-specific amplicon and the lowest number of reads was 24,341. This number of reads instilled confidence in catching all the richness present in our dataset, as the rarefaction curves showed that we were in the stationary phase of the curve. The read count matrix was transformed in relative abundance by dividing the reads affiliated to one taxa by the total number of reads in the sample (x/sum(x)), and a logarithmic transformation was also applied. To avoid the presence of 0 in the matrix, one artificial read was added to each cells of the data (Log10(x + 1)). Alpha diversity was analyzed using the Observed richness, Pielou’s evenness index, Shannon diversity index and the Inverted Simpson diversity index. Community composition was observed using barplot, jitter plot and krona plot, using the relative abundance data and heatmap with the Log10 transformed data. Statistical analyses were performed using a non-parametrical statistical test, as sequencing data did not fulfill the parametric test requirements (e.g., data normality and homoscedasticity, and independence of observation) using a *p*-value and False discovery rate threshold of 0.05. Kruskall–Wallis one-way analysis of variance was performed on the relative abundance sub-data of the main Synechococcus taxa to test if at least one taxa dominated the community. The hypothesis that the relative abundance distribution of the tested taxa are equal was the null hypothesis (H0) and the alternative hypothesis (H1) was that the relative abundance distribution of the tested taxa are not equal. If the Kruskall–Wallis test rejected H0, a post hoc test using the Conover–Iman squared rank test, with a Benjamini Yekutieli *p*-value adjustment procedure, was performed to determine which taxon or group of taxa dominate the community. The Benjamini Yekutieli procedure was chosen for *p* value adjustment because of the dependency between the taxa’s relative abundance observation. Barplots of the taxa of interest (*Planktothrix spp.*, *Microcystis spp.*, *Synechococcales*) and correlation observation of the main node from the network analysis were produced.

### 5.9. Toxin Extraction and Analysis

Toxins were extracted from filters using a methanolic extraction method and were, subsequently, analyzed via UPLC–MS/MS [24,65]. Briefly, 3 mL 50% (*v/v*) aqueous methanol was added to each filter and soaked for 30 min at room temperature. After vigorous mixing (vortex) for 10 min, samples were sonicated in an ultrasonic bath for 15 min, before centrifugation at 4000 × *g* for 10 min. The supernatant was collected in a separate tube and the above steps, excluding the initial 30 min soaking time, were repeated twice with the remaining pellet. The pooled supernatant was dried overnight using a speed vacuum system (Univapo 100H) and resuspended in 250 µL 50% aqueous methanol. Toxin samples were stored in glass vials at −20 °C until analysis.

Concentrations of different MCs were measured via UPLC–MS/MS, with an internal standard containing deuterated MC-LR and MC-LF (D_5_-MC-LF and D_7_-MC-LR) [24,65,87]. Three different MCs, MC-RR, MC-YR and MC-LR were used as external standards for analysis, with final concentrations of 2, 10 and 100 ng/mL. An Acquity H-class liquid chromatograph and a Waters XEVO TQ-S mass spectrometer were used. For UPLC, an Acquity BEH C18 1.7 µm column (2.1 × 50 mm) with a corresponding guard column, each kept at 40 °C, was used. Solvents A and B were composed of 10% and 90% acetonitrile, respectively, 100 mM formic acid and 6 mM NH_3_. Initial conditions were 25% B, held for 30 s, then 45% B within 30 s, 60% B within 180 s and 99% B within 12 s, which was held again for 30 s. The flow rate was 0.4 mL/min. Prior to application of the next sample, the column was re-equilibrated to 25% B over 78 s and held for 60 s. Injection volume was 5 µL. As described in Altaner et al., 2019, simultaneous analysis of MC congeners was carried out using five analysis windows, maximizing the scan time for each congener [65].

### 5.10. Evaluation of bbe Moldaenke FluoroProbe Data

For data acquisition, the Moldaenke FluoroProbe (FP) was slowly lowered (approximately 0.2 m/s) from the water surface to 100 m depth using a winch, then quickly pulled up again, while collecting data both ways. FP data was preprocessed and later plotted in 3D plots using MATLAB R2020a [88]. The datasets were chopped above 1 m depth and below 100 m depth and subsequently sorted. Data were interpolated to 0.1 m steps, and 570 nm fluorescence measurements and cryptophyta content (µg/L) data were extracted. Plotting was carried out using the MATLAB standard functions *surf* (3D) and *plot* (2D), and peaks were analyzed using *max*.

## Figures and Tables

**Table 1:** Primers used in this study.

Name of Forward and Reverse Primers	Sequence (5′-3′)	Target	Reference
HEPF	TTTGGGGTTAACTTTTTTGGGCATAGTC	*mcyE*	Jungblut and Neilan, 2006
HEPR	AATTCTTGAGGCTGTAAATCGGGTTT		
16S rDNA PTX fw	ATCCAAGTCTGCTGTTAAAGA	16S rDNA (only *Planktothrix* spp.)	Ostermaier and Kurmayer, 2009
16S rDNA PTX rv	CTCTGCCCCTACTACACTCTAG		
16S rDNA PTX TaqMan ^1^	AAAGGCAGTGGAAACTGGAAG		
mcyBA1 PTX fw	ATTGCCGTTATCTCAAGCGAG	*mcyBA1* (only *Planktothrix* spp.)	Ostermaier and Kurmayer, 2009
mcyBA1 PTX rv	TGCTGAAAAAACTGCTGCATTAA		
mcyBA1 PTX TaqMan ^1^	TTTTTGTGGAGGTGAAGCTCTTTCCTCTGA		
CYA359F	GGGGAATYTTCCGCAATGGG	16S rRNA (Cyanobacteria)	Nübel et al., 1997
CYA784R	ACTACWGGGGTATCTAATCCC		

^1^ TaqMan probes contain 5′ FAM (6-carboxyfluorescein, fluorescent reporter dye) and 3′ TAMRA (6-carboxy-tetramethylrhodamine, fluorescent quencher dye).

## Data Availability

The Illumina sequence data presented in this study is accessible at NCBI under the bioproject number PRJNA727470.

## Supplementary Materials

The following are available online at https://www.mdpi.com/article/10.3390/toxins13090666/s1, Figure S1: Common microcystin structure; Figure S2: Illustration of red colored filters; Figure S3: Relative abundance as quantified with qPCR; Figure S4: Localization of study site; Figure S5: Depiction of stable weather window prior to 1 July 2019; Figure S6: Depiction of depth profiles for 2009–2020; Figures S7 and S8: Mean wind speed and directions around the study site; Table S1: Sampling dates with corresponding sampling depths; Table S2: Assignment of ASVs with SILVA and Greengenes databases; Table S3: NCBI megablast of *C. gracile* and *S. rubescens* in 2019 and 2020; Table S4: Relative abundance of *Synechococcus* ASVs per date in 2019 and 2020; Table S5: Conover–Iman test of *Synechococcus* ASVs in 2019 and 2020.
