# Supplementary material for: Is Toxin-Producing Planktothrix sp. an Emerging Species in Lake Constance?
Source: Toxins (Basel). 2021 Sep 17;13(9):666. doi: 10.3390/toxins13090666 (PMC8472890; doi:10.3390/toxins13090666)
Supplement: Supplementary file 1 [file toxins-13-00666-s001.zip › toxins-1330408-supplementary.pdf]

Supplementary File:

Is Toxin-Producing *Planktothrix* sp. an Emerging Species in Lake Constance?

Corentin Fournier[2*] & Eva Riehle[1*], Daniel R. Dietrich[1#] and David Schleheck[2,3#]

[1]Human and Environmental Toxicology – University of Konstanz
[2]Microbial Ecology and Limnic Microbiology – University of Konstanz
[3]Limnological Institute – University of Konstanz
*shared 1st authorship
#shared senior authorship, correspondence: daniel.dietrich@uni-konstanz.de (DRD), david.schleheck@uni-konstanz.de (DS)

Figure S1

Common Microcystin (MC) monocyclic structure with a molecular weight of approx. 1 kDa, composed of seven amino acids, amongst which are three *D*-amino acids, a N-methyldehydroalanine, two *L*-amino acids at the hypervariable positions 2 and 4 (marked with red Z and X) and the unique amino acid ADDA (figure adapted from [1]).

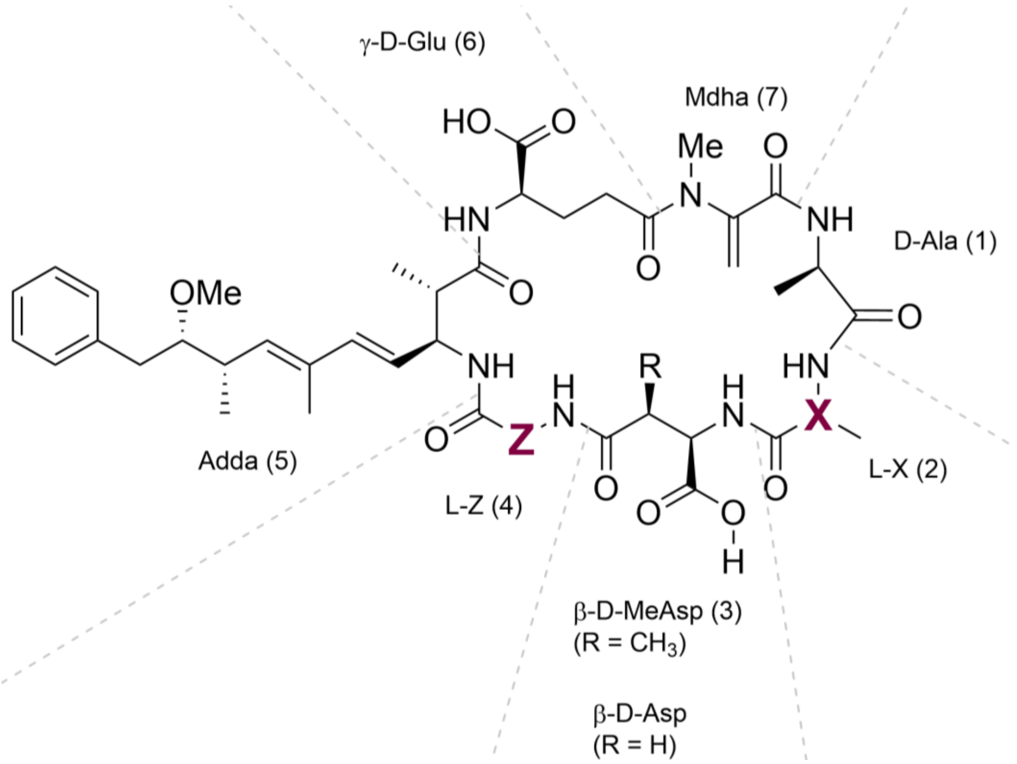

Figure S2

Representative illustration of the different coloration of glass fiber filters loaded with biomass collected from chlorophyll-a maximum (left) and DRM (right). Two liters of water taken at 4 m or 20 m water depth, respectively, were filtered through GF6 filters. The samples were taken on June 9th, 2021.

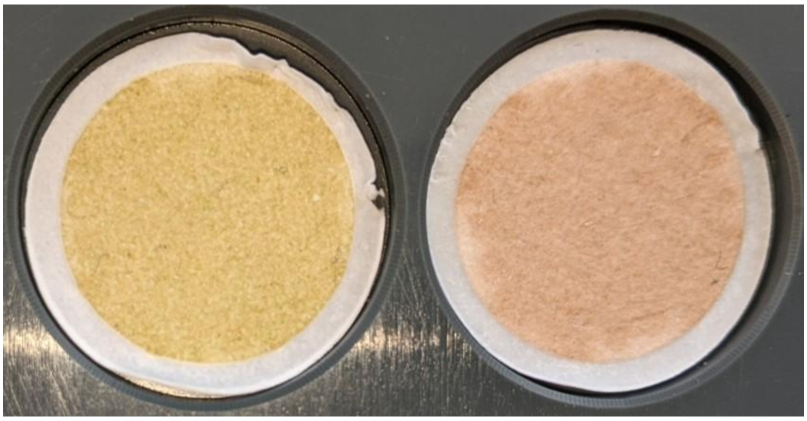

Figure S3

Relative abundance of *Planktothrix* spp. as determined by quantitative PCR. A *Planktothrix* 16S rDNA gene fragment and a *mcyBA1* domain were detected using the primer pairs from Ostermaier et al., 2009 and analysed as described in Materials and Methods section. Squares represent relative abundance of *Planktothrix*-specific 16S rDNA gene fragments and triangles represent *Planktothrix*-specific *mcyBA1*, which amplified the first adenylation domain of *mcyB*, one part of the microcystin gene cluster [2]. 2019 data are represented as single determinations, while 2020 data are represented as mean of biological triplicates (n = 3) ± SEM.

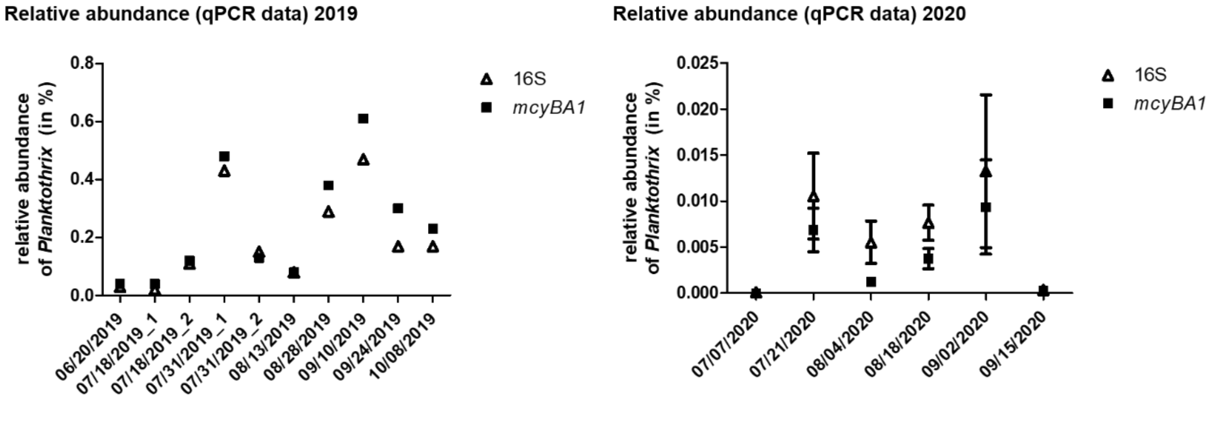

Figure S4

Localization of the study site in the Überlingen embayment of Lake Constance. The map was modified using the online tool UDO and is courtesy of LUBW Baden-Wuerttemberg [3]. The study site and the corresponding coordinates (47.757°N 9.1273°E) are indicated on the map.

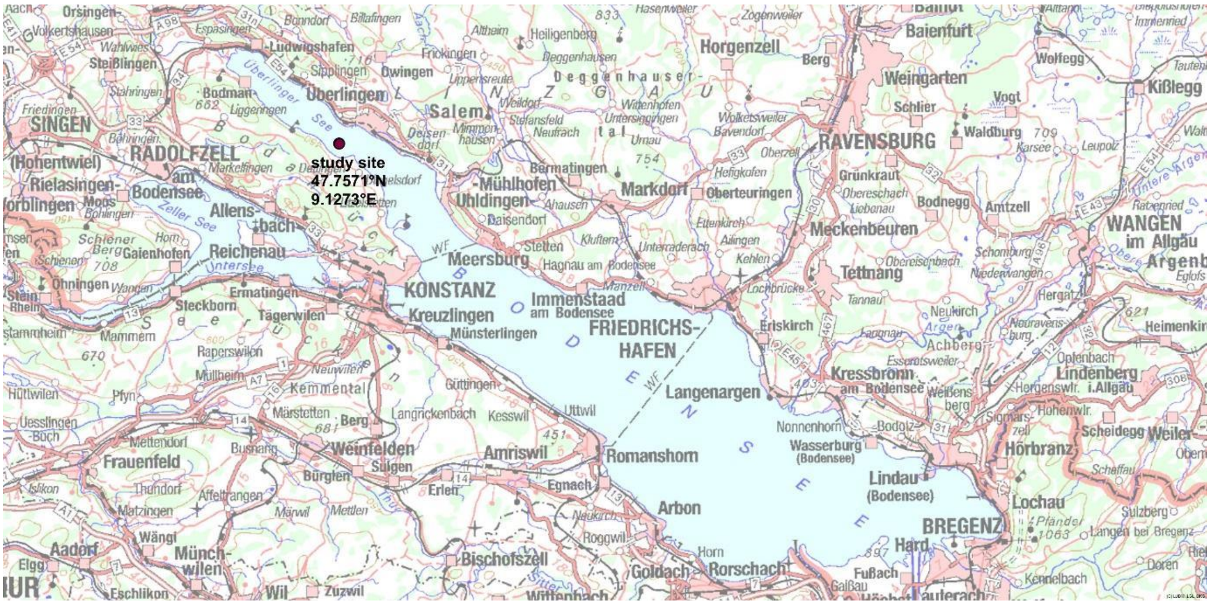

Figure S5

Detailed analysis of weather data in 2019. A: 3D plot of cryptophyta content (µg/L) from Jan-Dec of 2019, DRM is indicated by asterisk and marks the depth of maximal red pigment abundance (FluoroProbe 'cryptophyta' abundance, see main text); B: detailed analysis of 07/01/2019 FluoroProbe data, green bar indicates Secchi depth, red dotted bar indicates DRM depth; C: weather datasets six weeks prior and two weeks after the peak of red pigment abundance, including wind speed (m/s, black dotted line), mean temperature (°C, red line) and precipitation (mm, blue bars); D: radiation datasets six weeks prior and two weeks after July 1st, 2019 including diffuse (cyan line) and global radiation (black line, J/cm²) and daily sunshine (h, pink dotted line). Chocolate colored bars in C and D indicate July 1st and the peak in cryptophyta concentration, stable weather period is indicated as black bar from June 17th to June 30th, 2019.

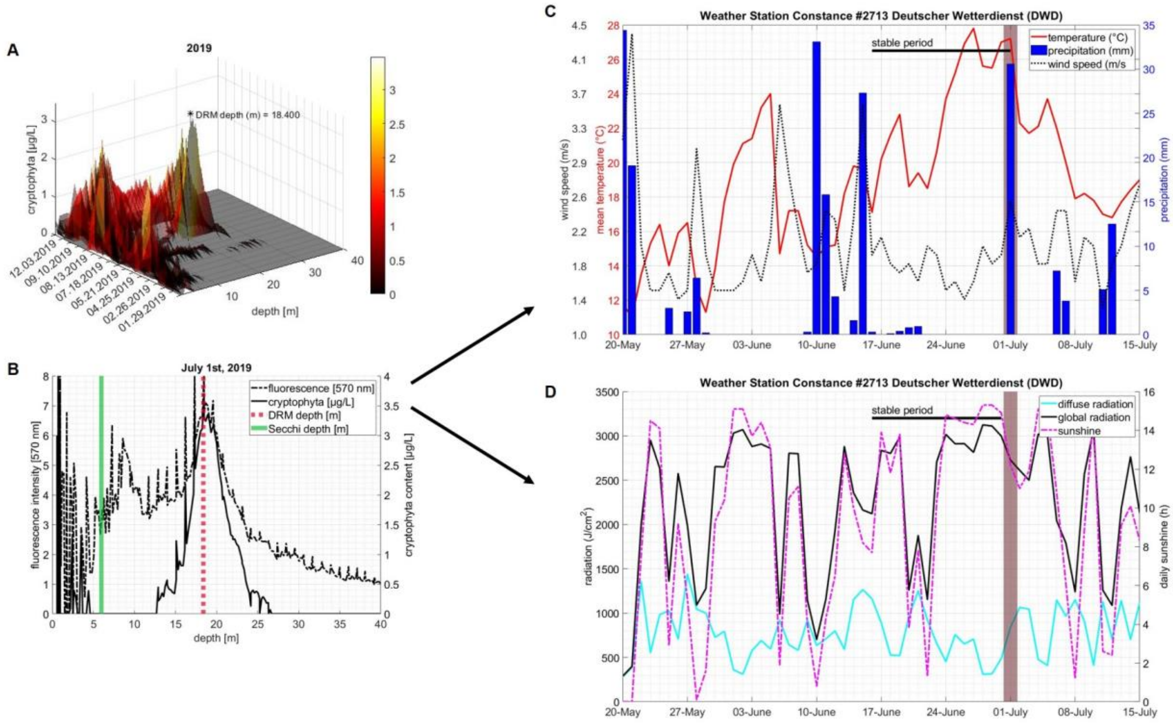

Figure S6

Depth-profiles recorded with the FluoroProbe from 2009 – 2020. Shown are the FluoroProbe profiles for 'cryptophyta' abundance recorded as proxy of red-pigment abundance in the water column from 0 – 40 m depth at the routine sampling site 'Wallhausen' in the Lake Überlingen embayment of Upper Lake Constance. Coordinates of the routine sampling site, 47.7571°N 9.1273°E. Note that in this figure the dates follow American-style date format.

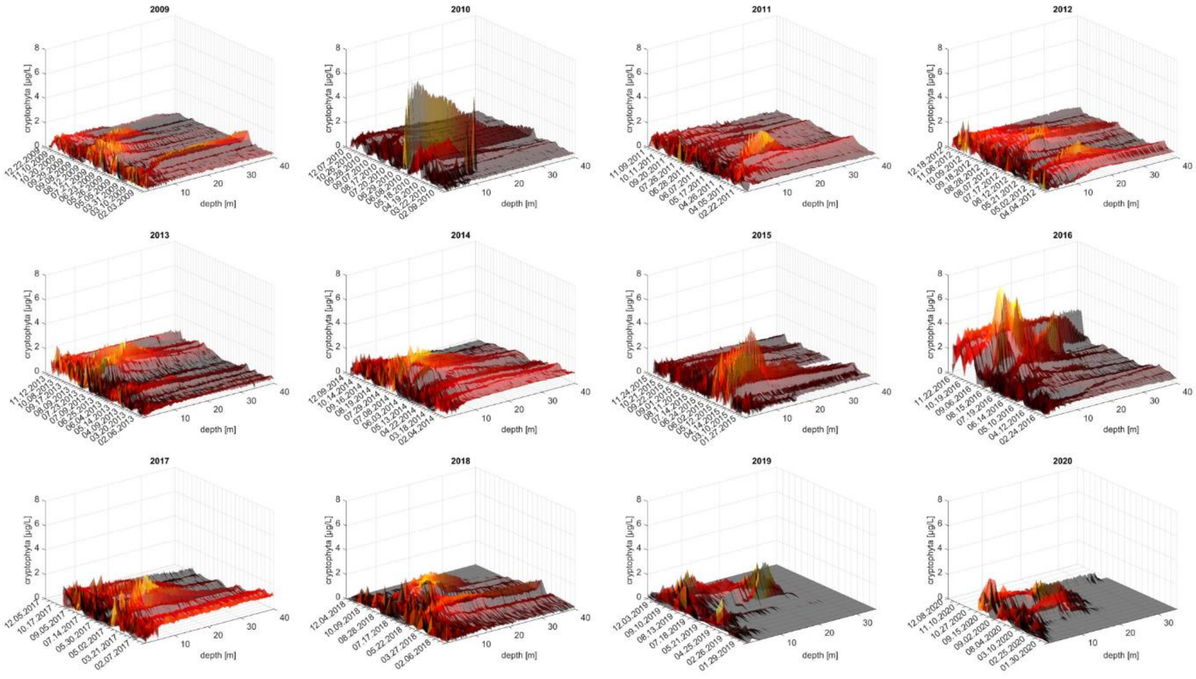

Figure S7

Mean wind speed and directions at three measurement locations near the study site. A: Illmensee wind station, B: Pfullendorf LUBW station, C: Singen LUBW station. Source: LUBW Baden-Wuerttemberg [3].

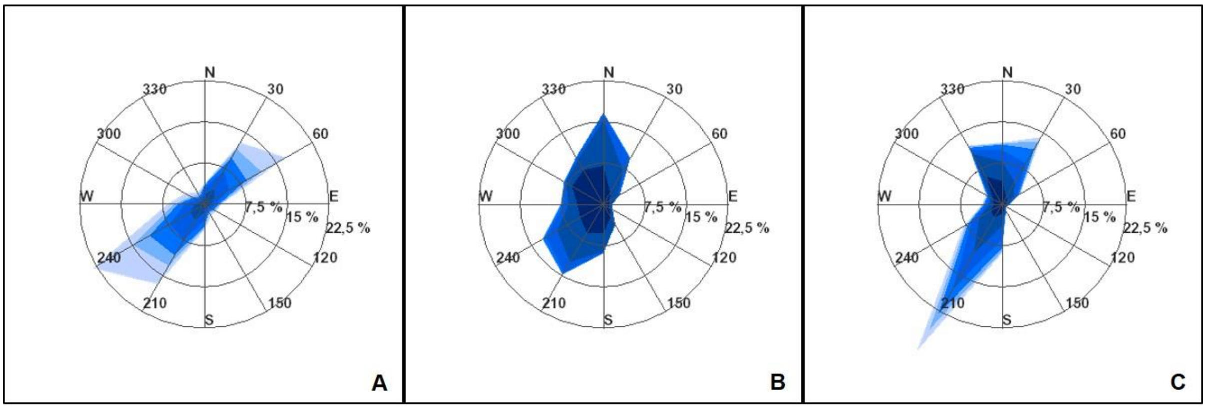

Figure S8

Overview map of mean wind speeds around the study site (47.757°N 9.1273°E). Data are courtesy of LUBW Baden-Wuerttemberg [3].

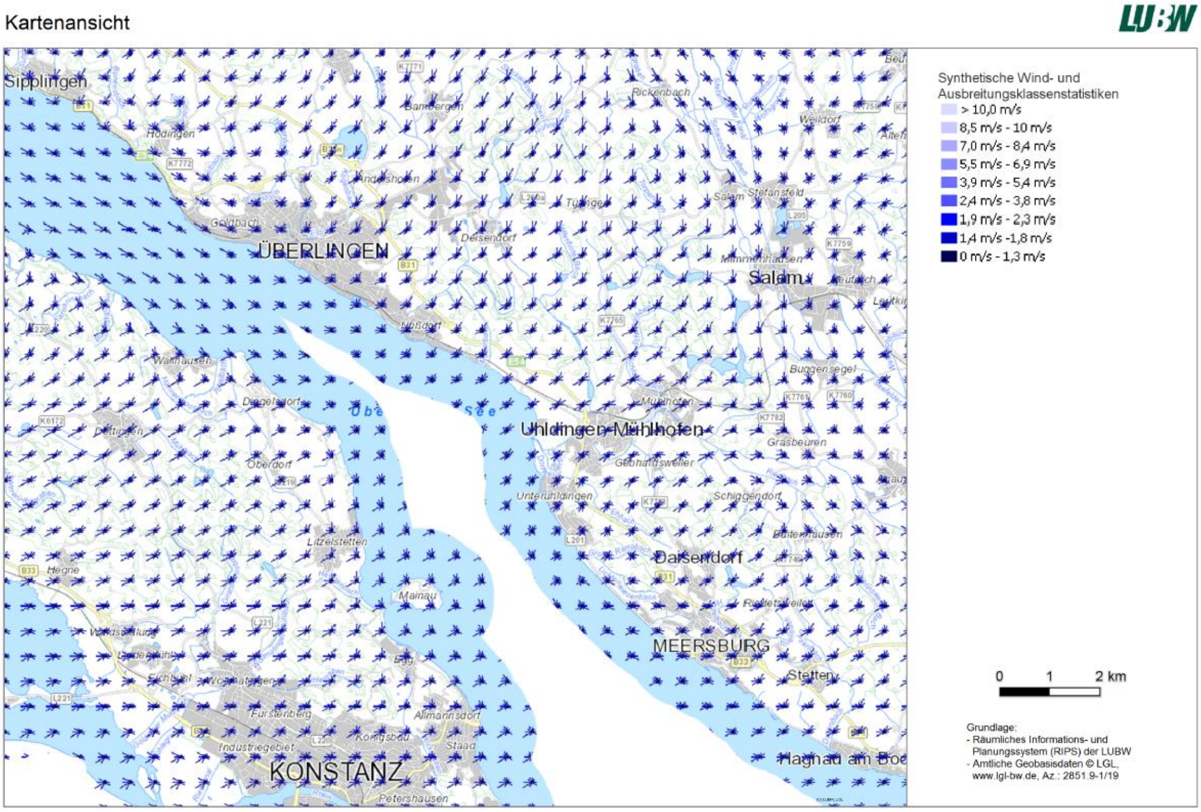

Table S1: Sampling dates with corresponding sampling depths

year	sampling date	sampling depth
2019	07/01/2019	18.5 m
	07/18/2019	16 m
	07/31/2019	20 m
	08/13/2019	17.5 - 18.5 m
	08/28/2019	23.3 - 25.5 m
	09/10/2019	7.5 m
	09/24/2019	N/A
	10/08/2019	10 m

year	sampling date	sampling depth
2020	07/07/2020	20 m
	07/21/2020	12.5 m
	08/04/2020	18 m
	08/18/2020	15 m
	09/02/2020	17 m
	09/15/2020	N/A
	10/27/2020	8 m
	10/27/2020	25 m
	11/10/2020	8 m
	12/08/2020	4 m

Table S2: Assignment of ASVs with SILVA and Greengenes databases

		SILVA_138					Greengenes				
	ASVs	Class	Order	Family.Lineage	Genus.Clade	Species.tribe	Class	Order	Family.Lineage	Genus.Clade	Species.tribe
2019	ASV1	Cyanobacteria	Cyanobacteriales	Microcystaceae	Microcystis_PCC-7914	Radiocystis_sp._JJ30-12	Oscillatoriophycideae	Chroococcales	Microcystaceae	Microcystis	NA
	ASV8	Cyanobacteria	Cyanobacteriales	Phormidiaceae	Planktothrix_NIVA-CYA_15	NA	Oscillatoriophycideae	Oscillatoriales	Phormidiaceae	Planktothrix	NA
	ASV9	Cyanobacteria	Cyanobacteriales	Phormidiaceae	Planktothrix_NIVA-CYA_15	Planktothrix_rubescens_CCAP_1459/14	Oscillatoriophycideae	Oscillatoriales	Phormidiaceae	Planktothrix	NA
	ASV13	Cyanobacteria	Synechococcales	Cyanobiaceae	Cyanobium_PCC-6307	NA	Synechococcophycideae	Synechococcales	Synechococcaceae	Synechococcus	NA
	ASV14	Cyanobacteria	Synechococcales	Cyanobiaceae	Cyanobium_PCC-6307	NA	Synechococcophycideae	Synechococcales	Synechococcaceae	Synechococcus	NA
	ASV15	Cyanobacteria	Synechococcales	Cyanobiaceae	Cyanobium_PCC-6307	NA	Synechococcophycideae	Synechococcales	Synechococcaceae	Synechococcus	NA
	ASV17	Cyanobacteria	Synechococcales	Cyanobiaceae	Cyanobium_PCC-6307	NA	Synechococcophycideae	Synechococcales	Synechococcaceae	Synechococcus	NA
	ASV18	Cyanobacteria	Synechococcales	Cyanobiaceae	Cyanobium_PCC-6307	NA	Synechococcophycideae	Synechococcales	Synechococcaceae	Synechococcus	NA
	ASV22	Cyanobacteria	Synechococcales	Cyanobiaceae	Cyanobium_PCC-6307	NA	Synechococcophycideae	Synechococcales	Synechococcaceae	Synechococcus	NA
	ASV28	Cyanobacteria	Synechococcales	Cyanobiaceae	Cyanobium_PCC-6307	uncultured_bacterium	Synechococcophycideae	Synechococcales	Synechococcaceae	Synechococcus	NA
	ASV29	Cyanobacteria	Synechococcales	Cyanobiaceae	Cyanobium_PCC-6307	uncultured_bacterium	Synechococcophycideae	Synechococcales	Synechococcaceae	Synechococcus	NA
	ASV30	Cyanobacteria	Synechococcales	Cyanobiaceae	Cyanobium_PCC-6307	uncultured_bacterium	Synechococcophycideae	Synechococcales	Synechococcaceae	Synechococcus	NA
	ASV33	Cyanobacteria	Synechococcales	Cyanobiaceae	Cyanobium_PCC-6307	NA	Synechococcophycideae	Synechococcales	Synechococcaceae	Synechococcus	NA

		SILVA_138					Greengenes				
	ASVs	Class	Order	Family.Lineage	Genus.Clade	Species.tribe	Class	Order	Family.Lineage	Genus.Clade	Species.tribe
2020	ASV4	Cyanobacteria	Synechococcales	Cyanobiaceae	Cyanobium_PCC-6307	NA	Synechococcophycideae	Synechococcales	Synechococcaceae	Synechococcus	NA
	ASV7	Cyanobacteria	Synechococcales	Cyanobiaceae	Cyanobium_PCC-6307	NA	Synechococcophycideae	Synechococcales	Synechococcaceae	Synechococcus	NA
	ASV8	Cyanobacteria	Synechococcales	Cyanobiaceae	Cyanobium_PCC-6307	uncultured_Synechococcus_sp.	Synechococcophycideae	Synechococcales	Synechococcaceae	Synechococcus	NA
	ASV10	Cyanobacteria	Synechococcales	Cyanobiaceae	Cyanobium_PCC-6307	NA	Synechococcophycideae	Synechococcales	Synechococcaceae	Synechococcus	NA
	ASV12	Cyanobacteria	Synechococcales	Cyanobiaceae	Cyanobium_PCC-6307	NA	Synechococcophycideae	Synechococcales	Synechococcaceae	Synechococcus	NA
	ASV17	Cyanobacteria	Synechococcales	Cyanobiaceae	Cyanobium_PCC-6307	uncultured_bacterium	Synechococcophycideae	Synechococcales	Synechococcaceae	Synechococcus	NA
	ASV21	Cyanobacteria	Synechococcales	Cyanobiaceae	Cyanobium_PCC-6307	uncultured_bacterium	Synechococcophycideae	Synechococcales	Synechococcaceae	Synechococcus	NA
	ASV22	Cyanobacteria	Synechococcales	Cyanobiaceae	Cyanobium_PCC-6307	uncultured_bacterium	Synechococcophycideae	Synechococcales	Synechococcaceae	Synechococcus	NA
	ASV25	Cyanobacteria	Synechococcales	Cyanobiaceae	Cyanobium_PCC-6307	NA	Synechococcophycideae	Synechococcales	Synechococcaceae	Synechococcus	NA
	ASV32	Cyanobacteria	Cyanobacteriales	Phormidiaceae	Planktothrix_NIVA-CYA_15	NA	Oscillatoriophycideae	Oscillatoriales	Phormidiaceae	Planktothrix	NA
	ASV33	Cyanobacteria	Cyanobacteriales	Phormidiaceae	Planktothrix_NIVA-CYA_15	Planktothrix_rubescens_CCAP_1459/14	Oscillatoriophycideae	Oscillatoriales	Phormidiaceae	Planktothrix	NA
	ASV34	Cyanobacteria	Cyanobacteriales	Microcystaceae	Microcystis_PCC-7914	NA	Oscillatoriophycideae	Chroococcales	Microcystaceae	Microcystis	NA

<u>Table S3a: NCBI megablast [4] results for *Cyanobium gracile* / *Synechococcus rubescens* affiliated sequences from 2019</u>

	ASVs	Description	Scientific Name	Max Score	Total Score	Query Cover	E value	Per. ident	Acc. Len	Accession
2019	ASV13	Synechococcus rubescens strain SAG 3.81 16S ribosomal RNA, partial sequence	Synechococcus rubescens	584	584	99%	1E-166	98.78	1456	NR_125481.1
		Cyanobium gracile PCC 6307 16S ribosomal RNA, partial sequence	Cyanobium gracile PCC 6307	529	529	99%	5E-150	95.73	1476	NR_102447.1
	ASV14	Synechococcus rubescens strain SAG 3.81 16S ribosomal RNA, partial sequence	Synechococcus rubescens	590	590	99%	2E-168	99.09	1456	NR_125481.1
		Cyanobium gracile PCC 6307 16S ribosomal RNA, partial sequence	Cyanobium gracile PCC 6307	529	529	99%	5E-150	95.73	1476	NR_102447.1
	ASV15	Synechococcus rubescens strain SAG 3.81 16S ribosomal RNA, partial sequence	Synechococcus rubescens	610	610	100%	2E-174	100	1456	NR_125481.1
		Cyanobium gracile PCC 6307 16S ribosomal RNA, partial sequence	Cyanobium gracile PCC 6307	521	521	100%	8E-148	95.15	1476	NR_102447.1
	ASV17	Synechococcus rubescens strain SAG 3.81 16S ribosomal RNA, partial sequence	Synechococcus rubescens	560	560	100%	2E-159	97.27	1456	NR_125481.1
		Cyanobium gracile PCC 6307 16S ribosomal RNA, partial sequence	Cyanobium gracile PCC 6307	538	538	100%	8E-153	96.06	1438	NR_114406.1
	ASV18	Cyanobium gracile PCC 6307 16S ribosomal RNA, partial sequence	Cyanobium gracile PCC 6307	560	560	100%	2E-159	97.27	1438	NR_114406.1
		Synechococcus rubescens strain SAG 3.81 16S ribosomal RNA, partial sequence	Synechococcus rubescens	560	560	100%	2E-159	97.27	1456	NR_125481.1
	ASV22	Cyanobium gracile PCC 6307 16S ribosomal RNA, partial sequence	Cyanobium gracile PCC 6307	588	588	100%	7E-168	98.79	1438	NR_114406.1
		Synechococcus rubescens strain SAG 3.81 16S ribosomal RNA, partial sequence	Synechococcus rubescens	510	510	100%	2E-144	94.55	1456	NR_125481.1
	ASV28	Cyanobium gracile PCC 6307 16S ribosomal RNA, partial sequence	Cyanobium gracile PCC 6307	577	577	100%	2E-164	98.18	1476	NR_102447.1
		Synechococcus rubescens strain SAG 3.81 16S ribosomal RNA, partial sequence	Synechococcus rubescens	532	532	100%	4E-151	95.76	1456	NR_125481.1
	ASV29	Cyanobium gracile PCC 6307 16S ribosomal RNA, partial sequence	Cyanobium gracile PCC 6307	566	566	100%	3E-161	97.58	1438	NR_114406.1
		Synechococcus rubescens strain SAG 3.81 16S ribosomal RNA, partial sequence	Synechococcus rubescens	544	544	100%	2E-154	96.36	1456	NR_125481.1
	ASV30	Cyanobium gracile PCC 6307 16S ribosomal RNA, partial sequence	Cyanobium gracile PCC 6307	577	577	100%	2E-164	98.18	1438	NR_114406.1
		Synechococcus rubescens strain SAG 3.81 16S ribosomal RNA, partial sequence	Synechococcus rubescens	538	538	100%	8E-153	96.06	1456	NR_125481.1
	ASV33	Cyanobium gracile PCC 6307 16S ribosomal RNA, partial sequence	Cyanobium gracile PCC 6307	549	549	100%	3E-156	96.67	1438	NR_114406.1
		Prochlorococcus marinus subsp. pastoris strain PCC 9511 16S ribosomal RNA, partial sequence	Prochlorococcus marinus subsp. pastoris	544	544	100%	2E-154	96.37	1465	NR_125480.1

Table S3b: NCBI megablast [4] results for *Cyanobium gracile* / *Synechococcus rubescens* affiliated sequences from 2020

	ASVs	Description	Scientific Name	Max Score	Total Score	Query Cover	E value	Per. ident	Acc. Len	Accession
2020	ASV4	Synechococcus rubescens strain SAG 3.81 16S ribosomal RNA, partial sequence	Synechococcus rubescens	706	706	100%	0	100	1456	NR_125481.1
		Cyanobium gracile PCC 6307 16S ribosomal RNA, partial sequence	Cyanobium gracile PCC 6307	617	617	100%	1E-176	95.81	1476	NR_102447.1
	ASV7	Synechococcus rubescens strain SAG 3.81 16S ribosomal RNA, partial sequence	Synechococcus rubescens	684	684	100%	0	98.95	1456	NR_125481.1
		Cyanobium gracile PCC 6307 16S ribosomal RNA, partial sequence	Cyanobium gracile PCC 6307	623	623	100%	2E-178	96.07	1476	NR_102447.1
	ASV8	Synechococcus rubescens strain SAG 3.81 16S ribosomal RNA, partial sequence	Synechococcus rubescens	678	678	100%	0	98.69	1456	NR_125481.1
		Cyanobium gracile PCC 6307 16S ribosomal RNA, partial sequence	Cyanobium gracile PCC 6307	623	623	100%	2E-178	96.07	1476	NR_102447.1
	ASV10	Cyanobium gracile PCC 6307 16S ribosomal RNA, partial sequence	Cyanobium gracile PCC 6307	656	656	100%	0	97.64	1476	NR_102447.1
		Synechococcus rubescens strain SAG 3.81 16S ribosomal RNA, partial sequence	Synechococcus rubescens	656	656	100%	0	97.64	1456	NR_125481.1
	ASV12	Cyanobium gracile PCC 6307 16S ribosomal RNA, partial sequence	Cyanobium gracile PCC 6307	640	640	100%	0	96.86	1476	NR_102447.1
		Prochlorococcus marinus subsp. pastoris strain PCC 9511 16S ribosomal RNA, partial sequence	Prochlorococcus marinus subsp. pastoris	628	628	100%	5E-180	96.34	1465	NR_125480.1
	ASV17	Cyanobium gracile PCC 6307 16S ribosomal RNA, partial sequence	Cyanobium gracile PCC 6307	684	684	100%	0	98.95	1438	NR_114406.1
		Synechococcus rubescens strain SAG 3.81 16S ribosomal RNA, partial sequence	Synechococcus rubescens	606	606	100%	2E-173	95.29	1456	NR_125481.1
	ASV21	Cyanobium gracile PCC 6307 16S ribosomal RNA, partial sequence	Cyanobium gracile PCC 6307	656	656	100%	0	97.64	1476	NR_102447.1
		Synechococcus rubescens strain SAG 3.81 16S ribosomal RNA, partial sequence	Synechococcus rubescens	634	634	100%	0	96.6	1456	NR_125481.1
	ASV22	Cyanobium gracile PCC 6307 16S ribosomal RNA, partial sequence	Cyanobium gracile PCC 6307	667	667	100%	0	98.17	1476	NR_102447.1
		Synechococcus rubescens strain SAG 3.81 16S ribosomal RNA, partial sequence	Synechococcus rubescens	623	623	100%	2E-178	96.07	1456	NR_125481.1
	ASV25	Synechococcus rubescens strain SAG 3.81 16S ribosomal RNA, partial sequence	Synechococcus rubescens	656	656	100%	0	97.64	1456	NR_125481.1
		Cyanobium gracile PCC 6307 16S ribosomal RNA, partial sequence	Cyanobium gracile PCC 6307	634	634	100%	0	96.6	1438	NR_114406.1

Table S4a: Relative abundance of *Synechococcus* ASVs in 2019

Relative abundance in percentage of the main *Synechococcus* taxa presented in Fig. 4 in 2019. The color scheme is equivalent of what is represented in the heatmap in Fig. 4A on the Log10 transformed data.

	01.07.2019	18.07.2019	31.07.2019	13.08.2019	28.08.2019	10.09.2019	24.09.2019	08.10.2019
**ASV13**	29.96	36.51	24.64	15.04	20.64	16.83	10.58	10.86
**ASV15**	11.47	16.34	22.17	22.30	18.76	21.78	16.68	15.29
**ASV14**	8.52	17.42	15.53	21.27	9.29	18.43	21.05	14.75
**ASV29**	15.64	6.23	10.33	4.87	27.54	9.52	6.69	15.86
**ASV28**	21.66	8.19	9.27	10.96	5.22	14.00	7.92	7.10
**ASV22**	0.00	0.22	0.79	3.25	3.55	1.74	14.54	16.15
**ASV30**	8.30	4.05	4.65	5.14	2.89	3.74	2.39	2.92
**ASV18**	0.69	2.03	2.07	4.21	1.07	4.05	7.11	4.71
**ASV33**	0.13	4.20	2.37	7.16	2.73	0.10	0.21	0.26
**ASV17**	1.85	2.61	1.83	1.20	1.90	2.24	1.03	1.35

Table S4b: Relative abundance of *Synechococcus* ASVs in 2020

Relative abundance in percentage of the main *Synechococcus* taxa presented in Fig. 4 in 2020. The color scheme is equivalent of what is represented in the heatmap in Fig. 4C on the Log10 transformed data.

	07.07.2020	21.07.2020	04.08.2020	18.08.2020	02.09.2020	15.09.2020
**ASV4**	35.36	40.57	56.21	67.61	49.61	23.16
**ASV7**	48.06	20.52	8.62	5.95	12.18	25.12
**ASV21**	0.97	10.00	8.88	16.01	13.84	19.42
**ASV22**	7.37	7.13	2.62	2.09	3.96	10.45
**ASV8**	3.30	5.01	3.47	1.84	3.82	6.76
**ASV10**	2.87	3.17	1.13	0.90	2.86	7.45
**ASV12**	0.53	5.64	7.62	0.56	0.94	0.70
**ASV17**	0.15	1.02	2.51	1.71	4.67	0.75
**ASV25**	0.32	1.35	1.03	0.98	1.17	1.67

Table S5a: Conover-Iman test on *Synechococcus* ASVs of 2019

Conover-Iman False Discovery rate results after Benjamini Yekutieli p-value correction for multiple comparisons between the main *Synechococcus* ASVs of 2019 presented in the article in Fig. 4A and 4B.

		ASV13	ASV15	ASV14	ASV29	ASV28	ASV22	ASV30	ASV18	ASV33	ASV17
	**ASV13**		1.0000	1.0000	0.1417	0.0569	**0.0000**	**0.0000**	**0.0000**	**0.0000**	**0.0000**
	**ASV15**			1.0000	0.1312	0.0503	**0.0000**	**0.0000**	**0.0000**	**0.0000**	**0.0000**
	**ASV14**				0.8084	0.3926	**0.0000**	**0.0001**	**0.0000**	**0.0000**	**0.0000**
**2019**	**ASV29**					1.0000	**0.0009**	**0.0117**	**0.0005**	**0.0000**	**0.0000**
	**ASV28**						**0.0031**	**0.0357**	**0.0017**	**0.0000**	**0.0000**
	**ASV22**							1.0000	1.0000	1.0000	0.7186
	**ASV30**								1.0000	0.2297	0.1330
	**ASV18**									1.0000	0.9717
	**ASV33**										1.0000
	**ASV17**										

Table S5b: Conover-Iman test on *Synechococcus* ASVs of 2020

Conover-Iman False Discovery rate results after Benjamini Yekutieli p-value correction for multiple comparisons between the main *Synechococcus* ASVs of 2020 presented in the article in Fig. 4C and 4D.

		ASV4	ASV7	ASV21	ASV22	ASV8	ASV10	ASV12	ASV17	ASV25
	**ASV4**		0.8320	0.0952	**0.0039**	**0.0009**	**0.0001**	**0.0000**	**0.0000**	**0.0000**
	**ASV7**			1.0000	0.1426	**0.0447**	**0.0029**	**0.0001**	**0.0001**	**0.0000**
	**ASV21**				1.0000	0.4315	0.0537	**0.0026**	**0.0022**	**0.0006**
**2020**	**ASV22**					1.0000	0.6496	0.0715	0.0548	**0.0187**
	**ASV8**						1.0000	0.2312	0.1822	0.0677
	**ASV10**							1.0000	1.0000	0.5650
	**ASV12**								1.0000	1.0000
	**ASV17**									1.0000
	**ASV25**									

References

1. Altaner, S.; Jaeger, S.; Fotler, R.; Zemskov, I.; Wittmann, V.; Schreiber, F.; Dietrich, D.R. Machine learning prediction of cyanobacterial toxin (microcystin) toxicodynamics in humans. *ALTEX* **2020**, *37*, 24–36, doi:10.14573/altex.1904031.
2. Ostermaier, V.; Kurmayer, R. Distribution and abundance of nontoxic mutants of cyanobacteria in lakes of the Alps. *Microb. Ecol.* **2009**, *58*, 323–333, doi:10.1007/s00248-009-9484-1.
3. Baden-Wuerttemberg Landesanstalt fuer Umwelt (LUBW) Umwelt-Daten und -Karten Online (UDO).
4. Altschul, S.F.; Gish, W.; Miller, W.; Myers, E.W.; Lipman, D.J. Basic local alignment search tool. *J. Mol. Biol.* **1990**, *215*, 403–410, doi:10.1016/S0022-2836(05)80360-2.
